# A LIFE COURSE APPROACH TO IDENTIFYING EVICTION LAWS AFFECTING OLDER ADULTS AND HISTORICALLY MARGINALIZED GROUPS

**DOI:** 10.1093/geroni/igad104.0158

**Published:** 2023-12-21

**Authors:** Sara Bybee, Sarah Canham, Nasser Sharareh, Fernando Wilson, Nathanael Player, Andrea Wallace

**Affiliations:** University of Utah, Salt Lake City, Utah, United States; University of Utah, Salt Lake City, Utah, United States; University of Utah Population Health Sciences, Salt Lake City, Utah, United States; University of Utah Population Health sciences, Salt Lake City, Utah, United States; Utah State Courts’ Self-Help Center, Salt Lake City, Utah, United States; University of Utah, Salt Lake City, Utah, United States

## Abstract

Alongside population aging, more older adults are becoming at risk of homelessness. Eviction—a type of housing instability in which a landlord initiates legal forced expulsion from a rental property—is one of the main drivers of homelessness. Evictions and homelessness disproportionately affect older adults, individuals of color, women, sexual and gender minority populations, veterans, and those with disabilities. Evictions can have devastating short- and long-term effects on tenants, and these negative consequences may be especially pronounced for individuals with multiple minoritized identities such as older LGBTQ+ adults. Older LGBTQ+ individuals face higher baseline rates of poor mental health, smoking, and health risk behaviors. In this study, key informant interviews (N=5) were utilized to identify state eviction and landlord-tenant laws that disproportionately affect older adults and other historically marginalized groups. Using a life course approach to understanding health disparities as they accumulate across the lifespan, data from LawAtlas—an online database allowing the examination of specific laws across the US—was cross-referenced with data from key informants to identify the following laws which may disproportionately affect older adults and other historically marginalized populations: nuisance property ordinances, source of income discrimination, state fair housing protections, and minimum notice-to-vacate laws. This presentation will describe how intentionally or unintentionally, these laws disproportionately affect older adults and other historically marginalized populations and may lead to negative eviction outcomes, homelessness, and poor physical and mental health. Identifying how such discriminatory laws can contribute to health disparities across the lifespan is necessary to achieve health equity.

